# Consanguineous Marriage and Intellectual and Developmental Disabilities among Arab Bedouins Children of the Negev Region in Southern Israel: A Pilot Study

**DOI:** 10.3389/fpubh.2014.00003

**Published:** 2014-01-28

**Authors:** Hassan Abu Saad, Salman Elbedour, Eyad Hallaq, Joav Merrick, Ariel Tenenbaum

**Affiliations:** ^1^Al-Qasemi Academic College of Education, Baqa Al-Gharbiyye, Israel; ^2^Negev Regional Center for Research Development, Beer-Sheva, Israel; ^3^KAYE Academic College of Education, Beer-Sheva, Israel; ^4^Department of Human Development and Psychoeducational Studies, School of Education, Howard University, Washington, DC, USA; ^5^Department of Psychology, Al-Quds University, Jerusalem, Israel; ^6^National Institute of Child Health and Human Development, Jerusalem, Israel; ^7^Health Services, Division for Intellectual and Developmental Disabilities, Ministry of Social Affairs and Social Services, Jerusalem, Israel; ^8^Division of Pediatrics, Hadassah-Hebrew University Medical Center, Mt Scopus Campus, Jerusalem, Israel; ^9^Kentucky Children’s Hospital, University of Kentucky College of Medicine, Lexington, KY, USA

**Keywords:** consanguineous marriage, intellectual and developmental disability, mental retardation, Bedouins’ health, consanguinity

## Introduction

In this article, we present data from two special education schools that serve the Arab Bedouin population in the Negev region in southern Israel. Data were collected on 221 children (53.8% female and 46.2% male) with moderate and severe intellectual and developmental disability (IDD) in order to assess the extent of consanguineous background in these children. Findings showed that 61.5% of all the participants were offspring of parents who were biologically related, both first and second cousins. Almost 70% of the participants were diagnosed with moderate IDD, 20% with severe IDD, and 10% diagnosed with other developmental disorders. It is recommended to further investigate this population for a more detailed history and specific genetic disorders for appropriate genetic counseling for those already married and also to focus public health efforts to decrease the rate of marriages between relatives.

Consanguinity is a well-known risk factor for genetic disorders, including diseases and syndromes that present with intellectual and developmental disabilities. This is due to autosomal recessive disorders and also other inherited disorders. The vast majority of the behavioral genetics studies, which for the most part have focused on twins and adoptees (1, 2) have been conducted in highly industrialized western societies such as the United States and Northern European countries.

Despite the important and influential research undertaken on the impact of genetics and consanguineous marriage and the extent to which public awareness has been raised by these findings, only a limited number of investigations have been carried out in collective, non-western societies (e.g., Africa, Asia, and the Middle East) and there is scant empirical evidence regarding the genetic influence of consanguineous marriage in these societies. In these societies, consanguineous marriage is a common feature. There is, for example, a long tradition of such marriages in Japan, India, Sudani tribes, and Arab societies (3–8). Consanguineous marriage is also commonplace in the population of about 200,000 Bedouin Arabs living in the Negev desert in the south of Israel.

Studies conducted in Indian rural and urban populations showed a higher frequency of consanguineous marriages in rural compared with urban communities (9, 10). One study (11) showed that about 50% of all marriages in the rural population were consanguineous marriages with 52.6% of these consanguineous marriages involving first cousin; whereas, in the urban area, consanguineous marriages accounted for about 30% of the total number of marriages, with 60.9% of these marriages involving first cousin relationships.

Research indicates that a large segment of the world population practices certain forms of “inbreeding.” According to Bittles (5), 20–50% of all marriages occur between biologically related people in parts of Central, South, and West Asia and North Africa. The most popular matches are between first cousins, double first cousins (where the spouses share both sets of grandparents), or uncles and nieces. Although less than 1% of marriages are consanguineous in North America and Europe, up to 10% of marriages in East and West Africa and South America are between kin. The percentages could also be high in rapidly growing populations in Middle Africa, the Caribbean, Central America, East Asia, and Southeast Asia, for which no reliable figures exist.

Changes in culture and the influence of the Western World also affect the rate of consanguineous marriage resulting in a decrease today (12). Among parents of 14,237 newborns in Bahrain in 2008–2009, the total consanguinity and first cousin marriage rates over a period of 4 months in 2008 were 10.9 and 6.9% respectively; while during all of 2009 the rates were 11.4 and 6.8% respectively (12). Compared with earlier data, first cousin marriage rates in Bahrain declined from 24% to nearly 7% (12).

## A Pilot Study

The pilot study was conducted in two special education schools in the south of Israel and included 221 children with moderate and severe intellectual disability (aged 6–18 years). The data collection was conducted via records from the schools on age, gender, genetic relation between parents, and the degree of intellectual disability.

In Israel, children are sent to special schools after standard assessment procedures and following the recommendations of local professional committees that review the children’s records, tests, and assessments. This study was performed with the approval of the IRB of the Negev Regional Center for Research Development, Beer-Sheva, Israel.

## Results

The sample size included all 221children enrolled in both schools, of them 119 (54%) were boys and 102 (46%) were girls. All children in these schools were diagnosed with intellectual disability and no borderline children were enrolled in both schools. Almost 70% (69.7%) of the children had moderate intellectual disability, about 20% (20.4%) severe, and 10% of the participants with other developmental disorder. One hundred thirty-six (61.5%) of these children were offspring of consanguineous marriages.

## Discussion

This initial screening clearly indicates that the vast majority of the study population was children of consanguineous couples. This puts the offspring’s health at risk and raises the question about the public awareness of the genetic risks of consanguineous marriages for cultural and socio-economic motives. Since this part of the Arab population (Arab Bedouins of the Negev) is a traditional population, it is important to work on public health efforts to decrease the incidence of consanguineous couples. As many of these marriages are set between the parents of the young couples, a better understanding of the possible consequences of marriage within the family may prevent such marriages to be set, hence preventing disorders in the offspring.

The findings of the present study are similar to findings from other studies (13, 14) indicating that intellectual and developmental disabilities in addition to other genetic disorders are most likely to occur among inbred offspring and the risk is significantly higher than in non-consanguineous families.

Although most researchers accept the causal role of genetics, the exact genetic link and how it operates needs more investigations. Researchers in Israel (14) identified a protein-truncating mutation, G408fsX437, in the gene CC2D1A on chromosome 19p13.12 in nine consanguineous Israeli Arab families with severe autosomal recessive disease and intellectual disability. The subjects tested were healthy women who were invited to undergo the genetic screening test as a part of their routine pregnancy monitoring and 117 reported a family history positive for intellectual disability. About 524 pregnant or pre-conceptional women were tested and found 47 carriers (approximately 1/11), whose spouses were then recommended to undergo testing. Eight carrier couples were identified, who were given genetic counseling and offered prenatal diagnosis. Of all the marriages, 28.6% were consanguineous; 16.5% of the total was between first cousins. The high prevalence of the mutation can be explained both by the founder effect owing to the generally high consanguinity rate among the inhabitants of the village, and also because two families with excessive numbers of offspring with intellectual disability were unacceptable as marriage partners by the rest of the families.

In Jordan (15), 20–30% of all marriages are cousin mating and 69% of these are first cousin. Among families of first cousin mating, about 30% had the highest rate of autosomal recessive conditions for different genetic disorders, such as sporadic undiagnosed cases of intellectual disability, congenital anomalies, and dimorphism in which they may have autosomal recessive etiology (15).

In Qatar (16), a study of 1,515 women found 54% consanguineous marriages and the most common form again between first cousins. Inbred children of these women had a significantly higher rate of asthma, intellectual disability, epilepsy, and diabetes compared with children of non-consanguineous couples.

A recent study (17) from Brazil in a house-to-house population-based survey in the state of Paraíba, 20,462 couples were interviewed regarding kinship relation, number of siblings, and offspring affected by intellectual or physical disabilities. The rate of consanguineous unions in the communities ranged from 6 to 41%. The overall average inbreeding coefficient (*F*) was 0.00602 ± 0.00253, ranging from 0.00134 to 0.01182. Communities situated on the backlands had an increased average value of *F* compared to those closer to the seashore (*P* = 0.024). The average rate of disabled offspring varied from 2.96 ± 0.68% for unrelated unions to 10.44 ± 16.86% for related couples at the level of double first cousins or uncle–niece.

The above research examples show that marriage in the family increases the risk of disability. The Bedouin Arabs of the Negev in southern Israel have practiced cousin-marriages for generations as a part of their cultural tradition. The exact rate of consanguineous marriages in this population is unknown. According to behavioral genetics theory, this type marriage puts the offspring at risk for a variety of genetically influenced disorders. Consequently, such children are likely to have lower cognitive ability including different degrees of IDDs in addition to genetic disease along with these disorders.

In conclusion, it is recommended to further investigate this population for a more detailed history and specific genetic disorders for appropriate genetic counseling for those already married and also to focus public health efforts to decrease the rate of marriages between relatives.
